# Abaxial Greening Phenotype in Hybrid Aspen

**DOI:** 10.3390/plants2020279

**Published:** 2013-04-24

**Authors:** Julia S. Nowak, Carl J. Douglas, Quentin C.B. Cronk

**Affiliations:** 1 Department of Biological Sciences, University of Toronto Scarborough, 1265 Military Trail, Toronto, ON, M1C 1A4, Canada; 2 Botany Department, University of British Columbia, 3529-6270 University Blvd., Vancouver, BC, V6T 1Z4, Canada; E-Mails: carl.douglas@ubc.ca (C.J.D.); quentin.cronk@ubc.ca (Q.C.B.C.)

**Keywords:** adaxial–abaxial polarity, *Populus trichocarpa*, *Populus tremula* x *tremuloides*, *ASYMMETRIC LEAVES1*, abaxial greenness, *KANADI*, leaf blade

## Abstract

The typical angiosperm leaf, as in *Arabidopsis*, is bifacial consisting of top (adaxial) and bottom (abaxial) surfaces readily distinguishable by the underlying cell type (palisade and spongy mesophyll, respectively). Species of the genus *Populus* have leaves that are either conventionally bifacial or isobilateral. Isobilateral leaves have palisade mesophyll on the top and bottom of the leaf, making the two sides virtually indistinguishable at the macroscopic level. In poplars this has been termed the “abaxial greening” phenotype. Previous work has implicated *ASYMMETRIC LEAVES1* (*AS1*) as an essential determinant of palisade mesophyll development. This gene, as well as other genes (84 in all) putatively involved in setting the dorsiventral axis of leaves, were investigated in two *Populus* species: black cottonwood (*Populus trichocarpa*) and hybrid aspen (*P. tremula* x *tremuloides*), representative of each leaf type (bifacial and isobilateral, respectively). Poplar orthologs of *AS1* have significantly higher expression in aspen leaf blade and lower in the petiole, suggestive of a potential role in the isobilateral leaf phenotype consistent with the previously observed phenotypes. Furthermore, an *ABERRANT TESTA SHAPE* (*ATS*) ortholog has significantly lower expression in aspen leaf tissue, also suggesting a possible contribution of this gene to abaxial greening.

## 1. Introduction

The genus *Populus* consists of approximately 29 species [1] containing two main types of leaves: bifacial and isobilateral [2]. Bifacial leaves are usually dark green on the adaxial surface and have a light-colored abaxial surface. These types of leaves are commonly associated with a rigid rounded petiole, which allows the adaxial surface of the leaf to be exposed to the sun. The primary photosynthetic tissues, the palisade mesophyll, are associated with the adaxial surface in bifacial leaves, while the abaxial surface consists of spongy mesophyll that allow the scatter of light due to air spaces and therefore contribute to the lighter coloration. Isobilateral leaves, on the other hand, are more commonly found within the genus [2] and are more or less uniformly green on both adaxial and abaxial leaf surfaces, in contrast to bifacial-leaved species. The petiole of these isobilateral leaves is mediolaterally flattened and unifacial, allowing the leaves to flutter in the wind [3]. Both surfaces of isobilateral leaves are strongly chlorophyllous and palisade or palisade-like mesophyll cells are present on both adaxial and abaxial surfaces. The development of the lower mesophyll cells is variable, from cells almost identical to upper or adaxial palisade mesophyll cells to less elongated palisade-like cells (also termed “abaxial palisade”). This “abaxial greening” or “abaxial greenness” phenotype [4] is thought to contribute to overall carbon gain due to a more even light distribution throughout the tree, improved carbon dioxide fixation following exposure to short sunflecks through the canopy, and decrease in leaf temperature [5,6,7].

### 1.1. Molecular Genetics of Leaf Variation

The genetic basis of abaxial greening phenotype and the associated unifacial petiole in *Populus* has been investigated [4], but the molecular genetic basis has not. The abaxial greening phenotype was mapped onto two major quantitative trait loci (QTLs) [4], but the genes responsible for this phenotype were not investigated further. A recent study [8], however, discussed the involvement of several genes in vegetative phase change in leaves of *Populus* x *canadensis* (*Populus nigra* x *deltoides*). Vegetative phase change or heteroblasty involves the transition from juvenile to adult leaf morphology. The differences in morphology are not always evident, but they are more pronounced when leaves transition from bifacial to isobilateral types during the life of a plant (e.g., *P.* x *canadensis*) [8]. In this hybrid, and other isobilateral-leaved poplars (comprising slightly over half of the genus [1]), juvenile leaves are bifacial while the adult leaves are isobilateral. Wang *et al*. [8] showed that expression levels of *squamosa protein binding-like* (*SPL*) genes and their interacting small RNAs are altered during this phase transition. Gene expression patterns contributing to the differences in leaf morphologies between juvenile bifacial and adult isobilateral leaves have not been investigated.

### 1.2. Objectives

This study was undertaken to determine differences in the patterns of gene expression of bifacial leaves of *Populus trichocarpa* and isobilateral leaves of *Populus tremula* x *tremuloides* (black cottonwood and hybrid aspen, respectively). Hybrid aspen (henceforth “aspen”, except where European aspen, *P. tremula*, is specified) is used in this study because it is a commercially grown tree and a convenient model organism. The parent species are very closely related (considered subspecies by some authors) and the leaves of the parent species do not differ significantly in anatomy. The genes responsible for palisade and spongy mesophyll development are unknown in poplar, but due to the association of the isobilateral blade with a unifacial petiole in aspen, possible candidate genes are those responsible for setting adaxial–abaxial or dorsiventral polarity in leaves. *ASYMMETRIC LEAVES1* (*AS1*) is one such gene involved in the dorsiventrality pathway. Loss-of-function (LOF) mutants in *AS1* and its orthologs (such as *PHANTASTICA* in *Antirrhinum*) [9] vary in the extent of the polarity defects they cause, from minimal in *Arabidopsis* to severe in *Antirrhinum*. LOF mutations of *AS1* orthologs in tomato and tobacco result in abaxialized phenotypes such as the development of a unifacial or abaxialized proximal region in leaves [10,11,12]. In tobacco, *AS1* is critical to adaxial patterning, particularly for the formation of palisade mesophyll [11]. *AS1* has also been implicated in vegetative phase change [13]. 

The objective of this study is, therefore, to sample a subset of candidate genes for dorsiventral polarity and investigate the overall differences in expression patterns between aspen and black cottonwood and their leaf blades, paving the way for a future study that can assess detailed expression patterns at the whole genome level and elucidate the genetic and developmental variation leading to the observed phenotypic differences in isobilateral and bifacial-leaved species. Since the aspen blade contains a greater abundance of those cell types that are characteristic of the adaxial surface (*i.e*., palisade mesophyll) in comparison to black cottonwood leaf blade, it can be predicted that aspen blade tissue will exhibit higher expression of some adaxial cell fate determining genes and/or lower expression of abaxial cell fate determining genes. The black cottonwood leaf blade has typical bifacial adaxial and abaxial anatomy, and therefore is expected to show standard adaxial–abaxial patterning and will be used a template for comparison. 

**Figure 1:**
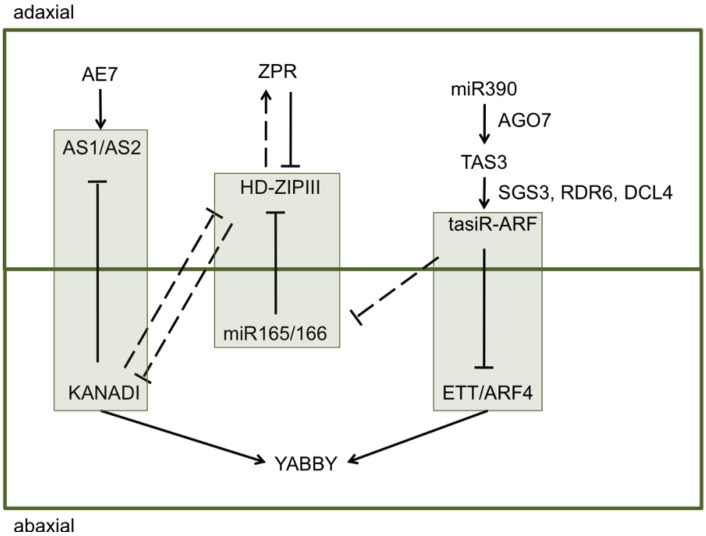
Adaxial–abaxial patterning in leaves is controlled by an array of transcription factors and corresponding small RNAs. Gene pathways enclosed with boxes, specify the major complexes setting dorsiventral polarity: (1) AS1/AS2-KANADI, (2) HD-ZIPIII-miR165/166, and (3) ETT/ARF4-tasiR-ARF. Solid lines indicate direct interactions while dashed lines indicate indirect interactions. Modified from Kidner and Timmermans [14].

In comparison to black cottonwood, aspen blade tissue may be expected to have higher expression of genes that are responsible for setting adaxial surface polarity due to the presence of adaxial type palisade cells at the abaxial side of the leaf and a decreased expression of some abaxial surface identity genes due to the absence of the spongy mesophyll cells at the abaxial surface. Marked overexpression of all adaxial identity genes (*i.e.*, *AE7* [*AS1/AS2 ENHANCER 7*], *AS1/AS2*, and others from Figure 1) would be unexpected due to the flatness of the leaf blade, which has clear and distinctive, dorsiventral polarity. If perturbations of the expression patterns of polarity genes were severe, the leaf blade would be malformed or unifacial either strongly adaxialized or abaxialized. A more likely scenario is therefore moderate differential expression of a small subset of abaxial and adaxial polarity genes in leaf blades between the two species. This is a scenario that can be tested against the results reported here.

## 2. Results and Discussion

### 2.1. Leaf Analysis

**Figure 2:**
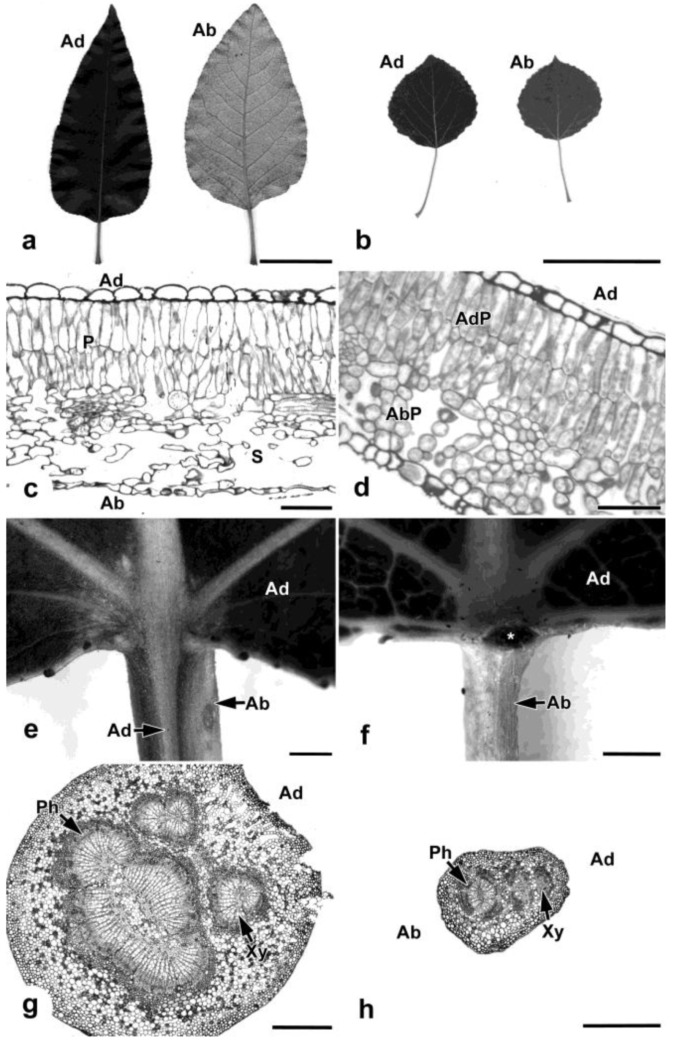
Black cottonwood (**a**,**c**,**e**,**g**) and aspen (**b**,**d**,**f**,**h**) leaf morphology and anatomy. (**a**) Black cottonwood leaves, showing dark green adaxial surface and lighter abaxialsurface. (**b**) Aspen leaves showing similarity in coloration of both adaxial and abaxial surfaces. (**c**) Transverse section of black cottonwood leaf blade showing palisade mesophyll at the adaxial surface and spongy mesophyll at the abaxial. (**d**) Transverse section of aspen leaf blade showing adaxial palisade mesophyll at the adaxial side and abaxial palisade or palisade-like mesophyll cells at the abaxial side. (**e**) Higher magnification of the adaxial side of the petiole/blade junction in black cottonwood leaf. The adaxial surface from the leaf blade is continued in the narrow region down the petiole, while the back and majority of the petiole consists of the abaxial surface. (**f**) Higher magnification of the adaxial side of the petiole/blade junction in aspen leaf. The adaxial surface of the leaf blade does not extend down the petiole, but is instead interrupted by the gland (asterisk) located at the “cross zone”. The petiole, therefore, consists mostly of the abaxial surface. (**g**) Transverse section of black cottonwood petiole with the adaxial surface labeled. The petiole contains three amphicribral vascular bundles. (**h**) Transverse section of aspen petiole with the adaxial and abaxial sides, labeled in relation to the shoot. There are two amphicribral vascular bundles within the petiole. Ad: adaxial surface, Ab: abaxial surface, P: palisade mesophyll, S: spongy mesophyll, AdP: adaxial palisade mesophyll, AbP: abaxial palisade mesophyll, Ph: phloem, Xy: xylem. Scale bars = 1 cm (**a**, **b**), 50 µm (**c**, **d**), 1 mm (**e**, **f**), 500 µm (**g**, **h**).

Black cottonwood and aspen are representative of the two major leaf variants in the genus *Populus*. Black cottonwood leaves are bifacial and contain palisade mesophyll tissues associated with the dark green adaxial side and spongy mesophyll associated with the lighter colored abaxial surface (Figure 2a,c). Aspen petioles are long relative to the leaf blade, with the petiole to total length ratio being smaller in black cottonwood leaves (0.17 ± 0.05) compared to aspen leaves (0.40 ± 0.06). Aspen leaves are isobilateral, exhibit the abaxial greening phenotype, and similar to black cottonwood contain adaxial palisade mesophyll cells, but unlike black cottonwood contain abaxial palisade mesophyll also in association with the abaxial side (Figure 2b,d).

In poplar, bifacial leaves are usually associated with rounded petioles and isobilateral with mediolaterally flattened petioles. Black cottonwood leaves have rounded petioles with a small region of adaxial surface that extends down the petiole (Figure 2e,g). Aspen leaves are associated with mediolaterally flattened petioles, which are bounded entirely by abaxial surface. The adaxial surface in aspen does not extend from the leaf down the petiole but ends at a “cross zone” at the petiole/blade junction where glands and small laminar outgrowths may be present (Figure 2f,h). Both species contain several amphicribral bundles (where phloem surrounds xylem) within the petioles (Figure 2g,h), indicative of abaxialization.

### 2.2. Transcriptome Data Analysis

A collection of 42 *Arabidopsis* genes was selected for study (Table 1, Table S1). These genes include those that have been implicated in adaxial–abaxial patterning (Figure 1) and vegetative phase change in *Arabidopsis* and poplar, including *AS1/AS2* and *SPL* orthologs. A large number of poplar candidate genes (84 in total) were initially selected for this study (Table S1) that are involved in the three complexes, including (1) AS1/AS2-KANADI, (2) HD-ZIPIII-miR165/166 (Class III HOMEODOMAIN-LEUCINE ZIPPER—microRNA165/166), and (3) ETT/ARF4-tasiR-ARF (ETTIN/AUXIN RESPONSE FACTOR4—trans-acting small interfering RNAs) (see for example 14 and 15 for more thorough reviews).

**Table 1:** *Arabidopsis* genes names and identified putative *P. trichocarpa* orthologs, including poplar gene id (for version 2.2 of the genome) and gene name for 18 poplar genes selected for detailed study, out of 84 initially screened. Gene function in *Arabidopsis*, from various references (cited within text or TAIR), is also presented.

Arabidopsis gene name	Arabidopsis accession number	Gene function in Arabidopsis	POPTR gene id (v2.2)	Poplar gene name
*AE7 (AS1/AS2 enhancer 7)*	AT1G68310	Adaxial polarity formation	*POPTR_0001s01820*	*Pt-AE7.1*
			*POPTR_0003s09670*	*Pt-AE7.2*
*AGO1 (argonaute 1)*	AT1G48410	Adaxial/abaxial cell fate specification; vegetative phase change	*POPTR_0012s03410*	*Pt-AGO1.1*
			*POPTR_0015s05550*	*Pt-AGO1.2*
*AS1 (asymmetric leaves 1)*	AT2G37630	Adaxial axis specification	*POPTR_0006s08610*	*Pt-AS1.1*
			*POPTR_0004s10250*	*Pt-AS1.2*
			*POPTR_0017s13950*	*Pt-AS1.3*
*AS2 (asymmetric leaves 2)*	AT1G65620	Adaxial axis specification	*POPTR_0010s18460*	*Pt-AS2.1*
			*POPTR_0008s07930*	*Pt-AS2.2*
*ATS (aberrant test shape)*	AT5G42630	Integument development; abaxial cell fate	*POPTR_0002s13170*	*Pt-ATS.1*
			*POPTR_0014s03650*	*Pt-ATS.2*
*YAB2 (YABBY 2)*	AT1G08465	Abaxial cell fate specification	*POPTR_0001s22180*	*Pt-YAB2.1*
			*POPTR_0127s00201*	*Pt-YAB2.2*
			*POPTR_0016s06760*	*Pt-YAB2.3*
*YAB3 (YABBY 3)*	AT4G00180	Abaxial cell fate specification	*POPTR_0003s11230*	*Pt-YAB3.1*
			*POPTR_0001s00240*	*Pt-YAB3.2*
*ZPR3 (little zipper 3)*	AT3G52770	Adaxial cell fate specification	*POPTR_0006s08320*	*Pt-ZPR3.1*
			*POPTR_0010s24410*	*Pt-ZPR3.2*

In order to access overall expression patterns of these genes, mean RPKM values (reads per kilobase of exon model per million reads mapped) for 84 selected genes in young leaf samples were calculated and expression levels of black cottonwood and European aspen (*P. tremula*) were compared. A number of genes (28 in total) showed significant differences in expression in leaves between the two species with *p*-values < 0.05, which may indicate candidate genes for investigating differences in leaf blade types between the two species. Using these genes as a guideline, other putative orthologs were included and used for further RT-PCR analysis (71 genes in total) in order to determine whether there was an indication of differential expression between the two species. Genes that were not included in further analysis were *Pt-CRC.1*, *Pt-CRC.2*, *Pt-INO.1*, *Pt-INO.2*, *Pt-SGS3.3*, *Pt-SGS3.4*, *Pt-SGS3.5*, *Pt-SGS3.6*, *Pt-SGS3.7*, *Pt-YUC.1*, *Pt-YUC.2*, *Pt-YUC2.1*, and *Pt-YUC2.2* due to low leaf expression levels (RPKM < 5).

### 2.3. Leaf Blade and Petiole qRT-PCR Gene Expression Patterns

Differential expression was confirmed by qRT-PCR. Several genes had significantly lower expression in aspen blade tissues compared to the blades of black cottonwood including: *Pt-AE7.2*, *Pt-AGO1.2*, *Pt-AS2.1*, *Pt-ATS.2*, *Pt-YAB2.1*, *Pt-ZPR3.1,* and *Pt-ZPR3.2* (Table 2, Table S3). *Pt-AS1.2* and *Pt-YAB3.2*, on the other hand, were significantly more highly expressed in aspen blades compared to black cottonwood (Table 2, Table S3). There were no significant differences in transcript abundance between the leaf blades of the two species in the remaining genes analyzed, with the exception of *Pt-SPL43.2* which had lower expression in aspen in comparison to black cottonwood (*p*-value = 0.012). 

**Table 2:** Genes showing significant difference in expression between black cottonwood and hybrid aspen/European aspen leaf blades according to transcriptome sequencing (mRNA-seq), RT-PCR, and qRT-PCR (with *p*-value presented for qRT-PCR) results.

POPTR gene id (v2.2)	Gene name	Hybrid or European aspen abundance in relation to black cottonwood			
		mRNA-seq	RT-PCR	qRT-PCR	qRT-PCR *p*-value
*POPTR_0003s09670*	*Pt-AE7.2*	-	-	-	0.020
*POPTR_0015s05550*	*Pt-AGO1.2*	NS	+	- *	0.008
*POPTR_0004s10250*	*Pt-AS1.2*	NS	NA	+ *	0.009
*POPTR_0010s18460*	*Pt-AS2.1*	-	-	-	0.028
*POPTR_0014s03650*	*Pt-ATS.2*	+	+	- *	0.030
*POPTR_0001s22180*	*Pt-YAB2.1*	-	NA	- *	0.010
*POPTR_0001s00240*	*Pt-YAB3.2*	+	+	+	0.00008
*POPTR_0006s08320*	*Pt-ZPR3.1*	-	-	-	0.020
*POPTR_0010s24410*	*Pt-ZPR3.2*	+	+	-	0.005

- denotes lower transcript abundance or expression level in hybrid aspen or European aspen (for mRNA-seq data) in comparison to black cottonwood; + denotes higher transcript abundance or expression level in hybrid aspen or European aspen; NS—not significant; NA—no data available; asterisk (*) denotes resultant gene expression patterns with qRT-PCR consistent with polarity scenarios discussed.

**Figure 3:**
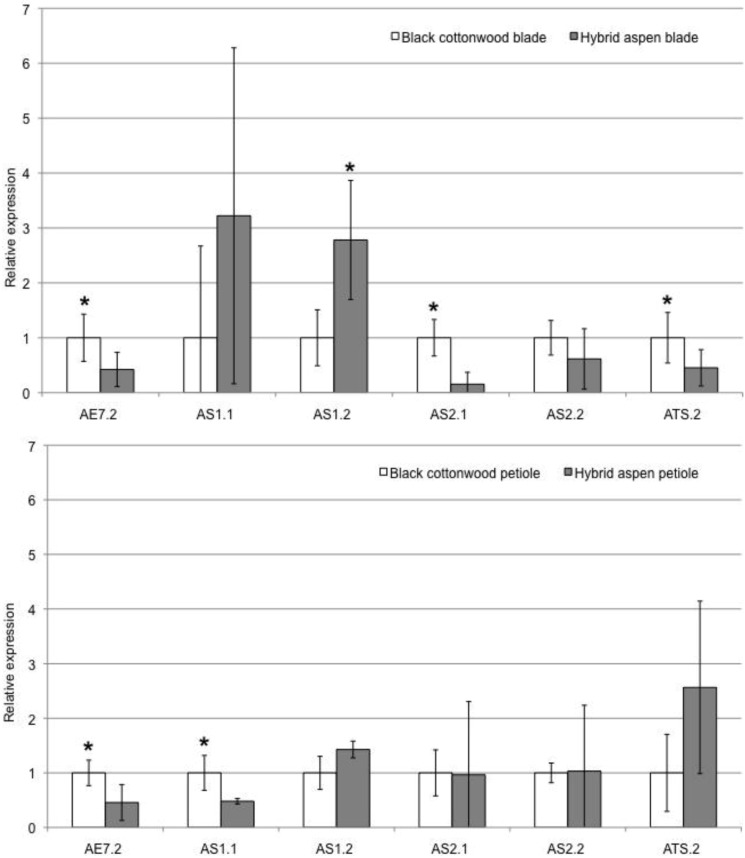
qRT-PCR results comparing black cottonwood and aspen leaf blade (top) and petiole (bottom) tissues. Asterisks indicate the genes that show a significant difference in expression between the two species, with the asterisk above the gene in the species that has higher expression. White bars show relative expression in black cottonwood, while grey bars show relative expression in aspen. Error bars indicate variability observed across biological and technical replicates.

Further analysis revealed that several genes (*i.e.*, *Pt-AGO7.4*, *Pt-RDR6.2*, *Pt-SPL4.1*, *Pt -AE7.2*) had significantly lower expression in petiole tissue in aspen compared to black cottonwood. *Pt-AE7.2* therefore has lower expression in both blade and petiole of aspen, and so this difference in expression may be general and regardless of tissue type (Figure 3). The remaining genes tested did not show a significant difference in transcript abundance between the petioles of the two species. *Pt-AS1.1* was significantly lower expressed in aspen petiole compared to black cottonwood (Figure 3). A recent study [16] of *AE7* (*AS1/AS2 ENHANCER7*) in *Arabidopsis* showed that it interacts with *AS1/AS2* and promotes adaxial identity. It is therefore not unexpected for this gene (*Pt-AE7.2*) to also be downregulated along with *Pt-AS2.1* in the blade and *Pt-AS1.1* in the petiole (Figure 3).

*Pt-AGO1.2*, *Pt-ATS.2*, and *Pt-ZPR3.2* expression patterns were not consistent between all analyses (*i.e.*, mRNA-seq, RT-PCR, and qRT-PCR). This was likely due to the tissue variability between leaf samples used in transcriptome sequencing and in RT-PCR, with the former analysis using leaves that are younger than used for the RT-PCR study. The differences between the PCR and transcriptome results could therefore be due to differences in developmental stage of the tissue. This variability could readily be checked in future studies by repeating these experiments on a developmental series (from developing leaf primordia to mature leaves) of black cottonwood and aspen blade and petiole tissues. Developmental variability has already been shown in some of these genes, with expression of *ATS* in *Arabidopsis* leaves (for example) increasing as leaves mature [17]. Where variability was observed between RT-PCR and qRT-PCR, results of the latter analysis are given more weight, due to their quantitative nature (e.g., *Pt-AGO1.2*, see Table 2).

### 2.4. Abaxial Determinants in Aspen

In accordance with a simple polarity scenario presented above, some of the genes responsible for abaxial cell fate specification (such as *KAN* or *YAB*) were hypothesized be downregulated in the leaf blades of aspen, but this was only observed in *Pt-ATS.2* and *Pt-YAB2.1*. 

*ATS* (*KAN4*) belongs to the *KANADI* gene family that is involved in setting abaxial polarity (Figure 1). This gene has not been reported to function in dorsiventral leaf polarity in *Arabidopsis*, but rather is known to determine polarity in ovule integuments [18,19] and to regulate flavonoid biosynthesis in developing seeds [20]. A recent study has reported an increase in *ATS/KAN4* transcript abundance in LOF *as1* mutants [21]. This is consistent with our results showing upregulation of *Pt-AS1.2* and downregulation of *Pt-ATS.2* in aspen blade tissues. 

Due to the lack of a documented function of *ATS* in leaves, high levels of expression such as those detected in both species, particularly with transcriptome sequencing, are rather unexpected. These results may be suggestive of a different function of *Pt-ATS.2*, in particular, in poplar as compared to *Arabidopsis*. *Pt-ATS.1* did not amplify in RT-PCR analysis, but transcriptome data suggests the absence of differential expression in the two species. 

Similar to *KANADI* genes, *YABBY* gene expression is restricted to the abaxial domain being responsible for abaxial surface identify specification (Figure 1) [22,23]. Four of the *YABBY* family members are expressed in *Arabidopsis* leaves (*AFO/FIL* [*ABNORMAL FLORAL ORGANS/FILAMENTOUS FLOWER*], *YAB2*, *YAB3*, and *YAB5*) with *AFO/FIL* and *YAB3* acting in a partially redundant manner and having their highest expression in leaves [22,24]. In *Arabidopsis*, *fil yab3* double mutants cause adaxialization of leaves, while gain-of-function (GOF) mutations cause the inverse, abaxialization of leaves [22,25,26]. Single LOF mutations do not show leaf polarity defects, but *fil* mutants produce radial floral organs [27]. The predicted downregulation of these genes would be similar to LOF mutations, possibly causing slight effects on leaf polarity, if any. The observed upregulation in aspen blade tissues of *Pt-YAB3.2* might be expected to lead to phenotypes suggestive of GOF mutations in *Arabidopsis*, but abaxialized leaf blades are not observed in aspen. Whatever the function and role of *Pt-YAB3.2* it should be noted that its putative paralog, *Pt-YAB3.1*, does not show significant expression differences between the two species in either qRT-PCR or transcriptome analysis. Furthermore, another *YABBY* gene (*Pt-YAB2.1*) has lower expression in aspen blade. *YAB2* is the least highly expressed *YABBY* gene in *Arabidopsis* leaves with expression patterns overlapping with other genes in the family (*i.e.*, *AFO/FIL* and *YAB3*) [22]. Its redundant function and low expression is a possible reason for lack of extensive studies of this gene. 

### 2.5. *ARGONAUTE1* in Aspen

*AGO1* expression is variable between aspen and black cottonwood. In the qPCR study, it had significantly lower expression in aspen. It is involved with the post-transcriptional regulation of multiple pathways, including RNA silencing and degradation via RNA-induced silencing complex (RISC) assembly [28,29,30]. *AGO1* is expressed throughout leaf primordia [31] and interacts with *AS1/AS2*. LOF *ago1* mutations can have a variety of effects, including abaxialized organs and defects in the meristem [30,32]. Most genes within the dorsiventral leaf polarity network are involved in setting either the adaxial or abaxial surface polarity in *Arabidopsis*. *AGO1*, on the other hand, appears to be responsible for both abaxial and adaxial cell fate determination in lateral organs [33]. Previous studies have reported its role in abaxial cell fate specification in leaves [28] and adaxial cell fate specification in petals [31]. Due to this variability in cell fate specification in *Arabidopsis*, it is reasonable to assume that in poplar, *Pt-AGO1* genes may be expressed in both the abaxial or adaxial domain in leaves. *AGO1* is also required for *KNOX* repression and juvenile leaf fate maintenance, by delaying vegetative phase change [34]. Functional studies are required to determine the precise role of these genes in poplar in order to predict their effect in the abaxial greening phenotype. 

### 2.6. *ASYMMETRIC LEAVES1* in Aspen

Genes that are responsible for adaxial polarity specification in *Arabidopsis* include *AE7*, *AS1*, *AS2*, and others (Figure 1). Some of these genes might be expected to be upregulated in aspen blade tissue, due to the presence of more adaxial-like cell types (*i.e.*, palisade mesophyll in the lower part of the leaf). The only gene that fits this criterion is *Pt-AS1.2*. 

The gene *AS1* (ortholog of *PHANTASTICA* in *Antirrhinum* and *ROUGH SHEATH2* in maize) has been shown in previous studies to be expressed throughout leaf primordia, and to be associated with *AS2* [35] to promote cell determinacy and repress *KNOX* (*KNOTTED-LIKE HOMEOBOX*), *ETT/ARF3*, *KANADI2*, and *YABBY5* genes [14,36,37,38]. In *Arabidopsis*, overexpression of *AS1* does not result in strong dorsiventral polarity defects. Instead, LOF mutants of *as1* develop downwardly curled asymmetric leaves without obvious loss of adaxial identity [14,37,39,40]. In other species, including tomato and *Antirrhinum*, LOF mutations in *as1* have more dramatic effects, causing radialization (abaxialization) of the petiole and even the lamina [9,10,11,12,40]. This is potentially consistent with lower expression of *Pt-AS1.1* transcript observed in unifacial aspen petioles. 

Chua *et al*. [13] showed a role for *AS1* in vegetative phase transition via an interaction with a bromodomain-containing protein *GTE6* (*GENERAL TRANSCRIPTION FACTOR GROUP E6*). *AS1* along with *GTE6* are upregulated in mature leaves as a plant transitions from the juvenile phase. The abaxial greening phenotype is characteristic of mature (but not seedling) leaf forms, and this is potentially consistent with the higher expression of *Pt-AS1.2* in the aspen leaf blade. Along with its contribution to proximal radialization and vegetative phase transition, *AS1* has been implicated in the determination of palisade mesophyll cells in tobacco [11]. Tobacco *AS1* ortholog *NsPHAN* is expressed in the middle mesophyll tissue or the tissue between adaxial palisade mesophyll and abaxial spongy mesophyll. Its cell-specific expression is essential to the production of cell divisions that promote palisade development in the mesophyll tissue associated with the adaxial surface [11]. The increased levels of *Pt-AS1.2* in aspen blade, therefore, can potentially explain the increased production of palisade cells at the adaxial and abaxial surfaces. 

Due to the expression patterns observed for genes encoding members of the AS1/AS2-KANADI complex, these genes are plausible candidate genes to be responsible for the abaxial greening phenotype in aspen. This possibility receives some support from the other differentially expressed genes that interact with *AS1*, either directly or indirectly, including *AE7*, which is an enhancer of *AS1/AS2* genes [16]. *AGO1* also interacts with *AS1/AS2* in the adaxial domain [33,34]. Genes of the *KANADI* family, to which *ATS* belongs, restrict the expression of *AS1/AS2* to the adaxial side of the leaf [14]. *KANADI* genes also promote *YABBY* expression [26] and the downregulation of *ATS* and *YAB2* orthologs are therefore consistent with an upregulation of *AS1* in aspen leaves. *ZPR* genes interact indirectly with *AS1/AS2* via their repression of *HD-ZIPIII* genes, which also indirectly repress *KANADI* genes in *Arabidopsis* [35,36,41,42,43].

## 3. Experimental Section

### 3.1. Leaf Analysis

Leaves of *P. trichocarpa* and *P. tremula* x *tremuloides* were collected from a collection at Totem Field (UBC) and imaged using a flatbed scanner. Whole leaves were also photographed using a Nikon stereomicroscope with a DS-Ri1 camera (Nikon Corp.). Leaf blade and petiole lengths were measured from several different trees in 129 and 75 replicate leaves of black cottonwood and aspen, respectively. Leaf blade pieces from the middle of the blade, on either side of the midvein, and petiole pieces (middle) were cut from fresh leaves, fixed in 70% FAA, embedded in LR White, sectioned, and photographed according to the methods described in Nowak *et al*. [44].

### 3.2. Gene Selection

*Arabidopsis thaliana* amino acid sequences (42 in total) were downloaded from TAIR [45] and were BLASTed (tBLASTn) using Phytozome [46] against *Populus trichocarpa* v2.2 genome. Final selection of homologous genes was based on the “Gene Ancestry” output results from Phytozome, which list all of the families (with genomes published on Phytozome, including *P. trichocarpa*) that contain the gene of interest, with the exception of *YABBY*, *KANADI*, and *HD-ZIPIII* orthologs that were identified using a phylogenomics approach [24]. A total of 84 *P. trichocarpa* genes were selected and used in further analyses, described below.

### 3.3. mRNA-seq Gene Expression Data Analysis

The transcriptomes of whole expanding and fully expanded leaves from three representative *P. trichocarpa* and four representative *P. tremula* (European aspen) individuals were sequenced using the Illumina GAII platform as part of a larger project. *P. tremula* (European aspen) RNA samples were sequenced rather than *P. tremula* x *tremuloides* (hybrid aspen), as this was the preferred tissue in the larger project. Hybrid aspen was used in all other gene expression analyses. Sequencing protocols are as described [47], but the short-read mapping was updated to version 2.2 of *Populus trichocarpa* genome using methods described in Geraldes *et al*. [47]. Mean and standard deviation values of normalized transcript expression levels (reads per kilobase of exon model per million reads mapped or RPKM) were calculated for each of the 84 genes identified (Table S2). RPKM data was derived from the sequenced reads for *P. trichocarpa* and *P. tremula* replicate samples (three and four replicates, respectively) for young expanding leaves. The mean values for each of the genes between black cottonwood and aspen were compared using a Student’s t-test. The number of genes for further study was narrowed to 71 with the following criteria: (1) genes that showed a significant difference in expression levels between the two species in leaf samples, (2) genes that had expression levels higher than 5 RPKM, and (3) the remaining putative orthologs of genes that satisfy criteria 1 and 2, even if their RPKM was less than 5. For example, if *Pt-AE3.1* showed RPKM > 5 and a difference in expression levels between black cottonwood and aspen, but *Pt-AE3.2* did not meet either or both of these criteria, both were included in further analyses.

### 3.4. Reverse Transcriptase PCR

Tissue from the first fully opened leaf on a branch was collected on three separate days over a three-week period from three separate trees for black cottonwood and aspen. Care was taken to sample at the same developmental stage for all samples. Several leaves (blade and petiole collected into separate samples) from each of the three trees were pooled into one sample (per collection day), frozen in liquid nitrogen, and analyzed further using following molecular techniques.

DNA was extracted from black cottonwood and aspen leaf tissues using a modified CTAB protocol [48]. RNA was extracted from each of these species from developing leaf blades and petioles (for tissue comparison) using Invitrogen PureLink Plant RNA Reagent (Burlington, Ontario, Canada). Both DNA and RNA concentrations were measured with a NanoDrop ND-1000 spectrophotometer (Thermo Scientific, Wilmington, DE, USA). Two microliters of RNA was further treated with DNase (TURBO DNA-free, Ambion, Mississauga, Ontario) from which cDNA was synthesized using a RevertAid H Minus First Strand cDNA Synthesis Kit (Fermentas, Burlington, Ontario, Canada). cDNA samples were diluted to similar concentrations, as measured with a NanoDrop spectrophotometer, for further RT-PCR analysis. For each of the 71 genes, primers were designed using Primer3 [49] to amplify 200–500 bp regions in *P. trichocarpa*. Polymerase chain reactions (PCR) were run with the following PCR program: 94 °C 3 min, (94 °C 30 s, 56 °C 40 s, 72 °C 1 min) × 36, 72 °C 1 min. Poplar translation initiation factor 5A (TIF5A), previously used by Ralph *et al*. [50], was used as a positive control in all PCR reactions. Each of the 71 primer pairs was run at least three times to determine expression patterns in leaf blade and petiole of black cottonwood and aspen. DNA of each of the species was amplified using these primers to assess whether the primer specificity was poor in contrast to the gene not being expressed. Negative controls were also included in each reaction, which excluded DNA or cDNA template. PCR products were run on 1–2% agarose gels (at 120 V for 30–60 min) to determine fragment sizes and estimate product abundance based on intensity of fragment staining. 

Genes that met the following criteria were analyzed further using quantitative RT-PCR (qRT-PCR): (1) RT-PCR products from RNA derived from aspen leaf blades higher putative expression based on staining intensity, compared to RT-PCR products from RNA derived from black cottonwood leaf blade samples or (2) differential expression between the two species in either leaf blade or petiole tissue samples. Genes that did not amplify either in blade or petiole aspen samples were not included in further analysis. In these cases, we postulate that the primers (derived from the *P. trichocarpa* genome sequence) were too divergent from the aspen sequences to support amplification, and rather than indicating of lack of expression (these results were not further investigated). 

Genes selected for further qRT-PCR analysis were labeled under the following categories, according to the RT-PCR results: (1) Higher expression in aspen blade, compared to black cottonwood, (2) Expression not higher in aspen blade, (3) No expression in black cottonwood blade, compared to expression present in aspen, and (4) No amplification of cDNA or DNA in either or both species. Some genes did not amplify, especially in aspen likely due to the lack of primer specificity (as primers were designed based on the *P. trichocarpa* reference genome sequence). 

### 3.5. Quantitative RT-PCR

Five samples, including black cottonwood blade and petiole, aspen blade and petiole (for tissue comparison), and negative control, were included in each qRT-PCR run for each of the genes. cDNA from three pooled biological replicate samples (described above in Section 3.4.) were all diluted to 1 ng/µL to start each qRT-PCR run with the same amount of starting template. The final cDNA concentration was measured using Qubit 2.0 fluorometer (Invitrogen), using the manufacturer’s protocol. Reactions were prepared using SsoFast EvaGreen Supermix protocol (Bio-Rad, Mississauga, Ontario) and were run using Bio-Rad iCycler iQ5 Real-Time PCR system with the following program: 95 °C 30 s (95 °C 5 s, 56 °C 5 s) × 40, 57 °C 5 s, 57 °C 5 s × 77. 

Expression or transcript abundance levels were presented as Cq values or the number of cycles when the template was used up [51]. The lower Cq value, the more template cDNA present and therefore more transcript present. Relative transcript abundance (normalized to TIF5A reference gene) for blade and petiole, separately, was calculated using the difference in Cq values or ∆Cq [Cq (test)—Cq (control)], which is the gene concentration compared to other control samples [52]. The “test” sample (as described in the ∆Cq formula) was designated as aspen as it was compared to the “control” or black cottonwood samples. The final results are graphed and presented as relative expression ratio: 2^-∆∆Cq [52]. 

## 4. Conclusions

Isobilateral leaves exhibit abaxial greening where, in its extreme form, the abaxial side of the leaf contains palisade-like mesophyll cells that are normally restricted to the adaxial side. A simple hypothesis is therefore that adaxial identity genes may be upregulated in such leaves, while there might be a reduction in abaxial gene expression relative to “normal” leaves. In this study, most genes that were analyzed had no significant differences in expression between the two species in leaf blades. However, a set of genes was found to be differentially expressed in aspen leaf blades in comparison to black cottonwood. In particular the upregulation of an adaxial identity gene *Pt-AS1.2* and the downregulation of abaxial cell fate determinants *Pt-ATS.2* and *Pt-YAB2.1* in aspen leaf blade, was consistent with this leaf polarity scenario. Similarly, downregulation of *Pt-AGO1.2* is interesting, as it is known to promote juvenile leaf fate maintenance, and therefore provides a possible link between leaf morphology and phase change in *Populus*. 

As aspen has abaxialized unifacial petioles, the finding of downregulation of *Pt-AS1.1* in aspen petiole is also consistent with the hypothesis. The two poplar *AS1* paralogs investigated show differential expression patterns between blade and petiole within a single leaf. This suggests a positional regulation of these paralogs within the leaf for organ determination.

The documented roles of *AS1* in petiole abaxialization, vegetative phase change, and promotion of palisade mesophyll development is consistent with a role for *AS1* in the abaxial greening phenotype of aspen. However, although the abaxial side of the aspen blade may contain cells that are characteristic of the adaxial domain in other species, the leaf blade maintains its fundamental dorsiventral polarity. Maintaining dorsiventrally flattened leaf blades in aspen, while still allowing for the development of adaxial cell types in the abaxial side of the leaf, is likely to be accomplished by relatively subtle changes in gene regulatory networks. *AS1* and its interacting factors, especially the *AS1-KANADI* antagonistic gene network, are here shown to be good candidates for this, worthy of further investigation.

## Supplementary Materials

**Table S1:** *Arabidopsis* genes names and identified putative *P. trichocarpa* orthologs, including poplar gene id and gene name. Gene function in *Arabidopsis*, from various references (cited within text or TAIR), is also presented.

Arabidopsis gene name	*Arabidopsis* accession number	Gene function in *Arabidopsis*	POPTR gene id (v2.2)	Poplar gene name
*AE3 (AS1/AS2 enhancer 3)*	AT5G05780	Adaxial leaf identity specification	POPTR_0008s06540	*Pt-AE3.1*
			POPTR_0008s06550	*Pt-AE3.2*
*AE7 (AS1/2 enhancer 7)*	AT1G68310	Adaxial polarity formation	POPTR_0001s01820	*Pt-AE7.1*
			POPTR_0003s09670	*Pt-AE7.2*
*AFO (abnormal flower organ)*	AT2G45190	Abaxial cell fate specification	POPTR_0014s06210	*Pt-AFO.1*
			POPTR_0002s14600	*Pt-AFO.2*
*AGO1 (argonaute 1)*	AT1G48410	Adaxial/abaxial cell fate specification; vegetative phase change	POPTR_0012s03410	*Pt-AGO1.1*
			POPTR_0015s05550	*Pt-AGO1.2*
*AGO7 (argonaute 7)*	AT1G69440	Regulation of vegetative phase change	POPTR_0009s00660	*Pt-AGO7.1*
			POPTR_0010s17100	*Pt-AGO7.4*
*AGO10 (argonaute 10)*	AT5G43810	Primary SAM specification; miRNA binding	POPTR_0008s15860	*Pt-AGO10.1*
			POPTR_0010s09150	*Pt-AGO10.2*
*AN3 (angustifolia 3)*	AT5G28640	Leaf development	POPTR_0013s04090	*Pt-AN3.1*
			POPTR_0019s02320	*Pt-AN3.2*
*ARF4 (auxin response factor 4)*	AT5G60450	Abaxial cell fate specification	POPTR_0009s01700	*Pt-ARF4.1*
*AS1 (asymmetric leaves 1)*	AT2G37630	Adaxial axis specification	POPTR_0006s08610	*Pt-AS1.1*
			POPTR_0004s10250	*Pt-AS1.2*
			POPTR_0017s13950	*Pt-AS1.3*
*AS2 (asymmetric leaves 2)*	AT1G65620	Adaxial axis specification	POPTR_0010s18460	*Pt-AS2.1*
			POPTR_0008s07930	*Pt-AS2.2*
*CNA (corona)*	AT1G52150	Adaxial identity determination; vascular histogenesis	POPTR_0003s04860	*Pt-ATHB.11*
			POPTR_0001s18930	*Pt-ATHB.12*
*ATS (aberrant test shape)*	AT5G42630	Integument development; abaxial cell fate	POPTR_0002s13170	*Pt-ATS.1*
			POPTR_0014s03650	*Pt-ATS.2*
*CRC (crabs claw)*	AT1G69180	Abaxial axis specification; floral meristem determinacy	POPTR_0008s09740	*Pt-CRC.1*
			POPTR_0010s16410	*Pt-CRC.2*
*DCL4 (dicer-like 4)*	AT5G20320	Vegetative phase change	POPTR_0006s20310	*Pt-DCL4.1*
*DUF59 (domain of unknown function 59)*	AT3G50845	Adaxial polarity formation (AE7-like)	POPTR_0005s12480	*Pt-DUF59.1*
*ETT (ettin)*	AT2G33860	Abaxial cell fate specification	POPTR_0004s04970	*Pt-ETT.1*
			POPTR_0011s05830	*Pt-ETT.2*
*ATHB8 (Arabidopsis thaliana homeobox 8)*	AT4G32880	Xylem development	POPTR_0018s08110	*Pt-HB1.5*
			POPTR_0006s25390	*Pt-HB1.6*
*REV (revoluta)*	AT5G60690	Adaxial axis specification; vascular pattern formation	POPTR_0004s22090	*Pt-HB1.7*
			POPTR_0009s01990	*Pt-HB1.8*
*HYL (hyponastic leaves 1)*	AT1G09700	Vegetative phase control (Li et al. 2012)	POPTR_0005s19650	*Pt-HYL1.1*
			POPTR_0002s11200	*Pt-HYL1.2*
*INO (inner-no-outer)*	AT1G23420	Abaxial cell fate specification; ovule development	POPTR_0008s19330	*Pt-INO.1*
			POPTR_0010s05220	*Pt-INO.2*
*KAN (KANADI 1)*	AT5G16560	Abaxial identity specification	POPTR_0017s02220	*Pt-KAN.1*
			POPTR_0004s08070	*Pt-KAN.2*
			POPTR_0015s05340	*Pt-KAN.3*
			POPTR_0012s03900	*Pt-KAN.4*
*KAN2, KAN3 (KANADI 2, KANADI3)*	AT1G32240 AT4G17695	Abaxial cell fate specification; ovule development	POPTR_0003s09490	*Pt-KAN2/3.1*
			POPTR_0001s02010	*Pt-KAN2/3.2*
*PHB (phabulosa)* *PHV (phavoluta)*	AT2G34710 AT1G30490	Adaxial cell fate specification	POPTR_0011s10070	*Pt-PHB.1*
			POPTR_0001s38120	*Pt-PHB.2*
*PGY1 (piggyback 1)*	AT2G27530	Adaxial pattern specification; AS1 enhancer	POPTR_0007s11880	*Pt-PGY1.1*
*PGY2 (piggyback 2)*	AT1G33140	Adaxial pattern specification; AS1 enhancer	POPTR_0001s45810	*Pt-PGY2.1*
			POPTR_0011s15170	*Pt-PGY2.2*
*PGY3 (piggyback 3)*	AT3G25520	Adaxial pattern specification	POPTR_0013s13220	*Pt-PGY3.1*
*RDR6 (RNA-dependent RNA polymerase 6)*	AT3G49500	Leaf development	POPTR_0006s26980	*Pt-RDR6.1*
			POPTR_0018s01670	*Pt-RDR6.2*
*SE (serrate)*	AT2G27100	Adaxial/abaxial pattern regulation	POPTR_0004s20730	*Pt-SE.1*
			POPTR_0009s16020	*Pt-SE.2*
*SGS3 (suppressor of gene silencing 3)*	AT5G23570	Vegetative phase change	POPTR_0019s00300	*Pt-SGS3.1*
			POPTR_0001s07410	*Pt-SGS3.2*
			POPTR_0001s07420	*Pt-SGS3.3*
			POPTR_0003s18660	*Pt-SGS3.4*
			POPTR_0003s18670	*Pt-SGS3.5*
			POPTR_0003s18680	*Pt-SGS3.6*
			POPTR_0003s18690	*Pt-SGS3.7*
			POPTR_0003s01530	*Pt-SGS3.8*
*SPL4 (squamosa promoter binding-like 4)*	AT1G53160	Vegetative phase change	POPTR_0001s40870	*Pt-SPL4.1*
			POPTR_0011s11770	*Pt-SPL4.2*
*SPL4, SPL3 (squamosa promoter binding-like 3)*	AT2G33810 (SPL3)	Vegetative phase change regulation	POPTR_0004s04630	*Pt-SPL43.1*
			POPTR_0011s05480	*Pt-SPL43.2*
*SPL9 (squamosa promoter binding-like 9)*	AT2G42200	Vegetative to reproductive phase change transition	POPTR_0016s04890	*Pt-SPL9.1*
*YAB2 (YABBY 2)*	AT1G08465	Abaxial cell fate specification	POPTR_0001s22180	*Pt-YAB2.1*
			POPTR_0127s00201	*Pt-YAB2.2*
			POPTR_0016s06760	*Pt-YAB2.3*
*YAB3 (YABBY 3)*	AT4G00180	Abaxial cell fate specification	POPTR_0003s11230	*Pt-YAB3.1*
			POPTR_0001s00240	*Pt-YAB3.2*
*YAB5 (YABBY 5)*	AT2G26580	Abaxial cell fate specification	POPTR_0006s06700	*Pt-YAB5.1*
		Abaxial cell fate specification	POPTR_0018s12990	*Pt-YAB5.2*
*YUC (yucca)*	AT4G32540	Auxin biosynthesis; regulation of leaf development	POPTR_0006s26430	*Pt-YUC.2*
			POPTR_0018s01210	*Pt-YUC.1*
*YUC2 (yucca 2)*	AT4G13260	Auxin biosynthesis	POPTR_0006s26000	*Pt-YUC2.1*
			POPTR_0018s00840	*Pt-YUC2.2*
*ZPR1 (little zipper 1)*	AT2G45450	Adaxial cell fate specification; interacts with REV	POPTR_0003s11710	*Pt-ZPR1.1*
			POPTR_0001s08220	*Pt-ZPR1.2*
*ZPR2 (little zipper 2)*	AT3G60890	Adaxial cell fate specification	POPTR_0002s15060	*Pt-ZPR2.1*
			POPTR_0014s06690	*Pt-ZPR2.2*
*ZPR3 (little zipper 3)*	AT3G52770	Adaxial cell fate specification	POPTR_0006s08320	*Pt-ZPR3.1*
			POPTR_0010s24410	*Pt-ZPR3.2*

**Table S2:** Transcriptome data for leaf tissues comparing black cottonwood (Ptr) and hybrid aspen (Ptmx) expression levels (RPKM) analyzed using RT-PCR. Grey shading denotes genes that have higher expression (RPKM) in hybrid aspen, compared to black cottonwood. A significant difference in expression levels between two species is determined with *p*-value < 0.05 (underlined). Bolded genes indicate RPKM > 5 in either species. Asterisks (*) indicate genes that were analyzed further with qRT-PCR.

Gene id	Gene name	Ptr mean RPKM	Ptmx mean RPKM	*p*-value
**POPTR_0008s06540**	***Pt-AE3.1***	**57.44 ± 8.16**	**40.04 ± 6.03**	**0.0221**
**POPTR_0008s06550**	***Pt-AE3.2***	**130.53 ± 7.99**	**95.61 ± 12.10**	**0.0077**
**POPTR_0001s01820**	***Pt-AE7.1***	**14.13 ± 4.50**	**11.69 ± 0.74**	**0.3228**
**POPTR_0003s09670**	***Pt-AE7.2 ****	**26.16 ± 6.35**	**16.34 ± 1.34**	**0.0268**
**POPTR_0014s06210**	***Pt-AFO.1***	**98.67 ± 16.85**	**52.38 ± 25.72**	**0.0437**
**POPTR_0002s14600**	***Pt-AFO.2***	**115.90 ± 22.05**	**58.60 ± 24.51**	**0.0244**
**POPTR_0012s03410**	***Pt-AGO1.1***	**68.79 ± 21.07**	**80.39 ± 13.30**	**0.4085**
**POPTR_0015s05550**	***Pt-AGO1.2****	**32.07 ± 11.43**	**30.67 ± 6.36**	**0.8422**
**POPTR_0009s00660**	***Pt-AGO7.1***	**7.09 ± 2.27**	**7.05 ± 0.88**	**0.9738**
**POPTR_0010s17100**	***Pt-AGO7.4 ****	**29.99 ± 5.89**	**24.16 ± 14.50**	**0.5471**
**POPTR_0008s15860**	***Pt-AGO10.1 ****	**12.84 ± 5.27**	**21.34 ± 6.48**	**0.1241**
**POPTR_0010s09150**	***Pt-AGO10.2***	**30.49 ± 10.63**	**17.88 ± 8.20**	**0.1343**
**POPTR_0013s04090**	***Pt-AN3.1***	**44.23 ± 11.87**	**33.86 ± 14.80**	**0.3674**
**POPTR_0019s02320**	***Pt-AN3.2***	**94.75 ± 41.53**	**76.91 ± 43.04**	**0.6057**
**POPTR_0009s01700**	***Pt-ARF4.1***	**25.40 ± 9.95**	**24.31 ± 4.08**	**0.8477**
**POPTR_0006s08610**	***Pt-AS1.1 ****	**52.68 ± 7.63**	**82.05 ± 41.07**	**0.2857**
**POPTR_0004s10250**	***Pt-AS1.2 ****	**25.90 ± 13.53**	**7.36 ± 5.93**	**0.0544**
**POPTR_0017s13950**	***Pt-AS1.3***	**51.14 ± 9.22**	**40.39 ± 11.10**	**0.2334**
**POPTR_0010s18460**	***Pt-AS2.1 ****	**23.81 ± 5.22**	**9.81 ± 6.46**	**0.0282**
POPTR_0008s07930	*Pt-AS2.2 **	4.85 ± 0.24	1.07 ± 0.75	0.0004
**POPTR_0003s04860**	***Pt-ATHB.11***	**13.90 ± 4.42**	**16.57 ± 6.17**	**0.5555**
**POPTR_0001s18930**	***Pt-ATHB.12***	**14.90 ± 4.21**	**36.77 ± 12.95**	**0.0399**
**POPTR_0002s13170**	***Pt-ATS.1***	**5.59 ± 0.60**	**4.54 ± 2.29**	**0.4815**
**POPTR_0014s03650**	***Pt-ATS.2 ****	**13.96 ± 3.14**	**35.83 ± 7.50**	**0.0055**
POPTR_0008s09740	*Pt-CRC.1*	0.00	0.02 ± 0.03	0.2751
POPTR_0010s16410	*Pt-CRC.2*	0.00	0.00	n/a
POPTR_0006s20310	*Pt-DCL4.1*	2.54 ± 1.04	4.97 ± 1.47	0.0592
**POPTR_0005s12480**	***Pt-DUF59.1***	**23.32 ± 9.89**	**21.87 ± 3.62**	**0.7928**
**POPTR_0004s04970**	***Pt-ETT.1***	**17.98 ± 5.65**	**20.09 ± 2.29**	**0.5199**
**POPTR_0011s05830**	***Pt-ETT.2***	**6.33 ± 1.56**	**8.77 ± 2.76**	**0.2322**
**POPTR_0018s08110**	***Pt-HB1.5 (ATHB8)***	**6.76 ± 2.39**	**19.63 ± 6.46**	**0.0234**
**POPTR_0006s25390**	***Pt-HB1.6 (ATHB8)***	**32.29 ± 7.56**	**23.22 ± 3.89**	**0.0897**
**POPTR_0004s22090**	***Pt-HB1.7 (REV)***	**6.04 ± 2.20**	**10.26 ± 3.55**	**0.1330**
**POPTR_0009s01990**	***Pt-HB1.8 (REV)***	**25.92 ± 8.83**	**9.02 ± 1.62**	**0.0118**
**POPTR_0005s19650**	***Pt-HYL1.1***	**16.41 ± 5.81**	**19.42 ± 2.47**	**0.3851**
**POPTR_0002s11200**	***Pt-HYL1.2***	**5.71 ± 1.29**	**8.58 ± 0.69**	**0.0121**
POPTR_0008s19330	*Pt-INO.1*	0.01 ± 0.01	0.00	0.2856
POPTR_0010s05220	*Pt-INO.2*	0.01 ± 0.02	0.00	0.2856
**POPTR_0017s02220**	***Pt-KAN.1***	**4.80 ± 0.39**	**7.01 ± 5.89**	**0.5545**
**POPTR_0004s08070**	***Pt-KAN.2***	**15.22 ± 1.36**	**9.80 ± 0.67**	**0.0009**
**POPTR_0015s05340**	***Pt-KAN.3***	**2.61 ± 1.64**	**6.26 ± 1.94**	**0.0471**
POPTR_0012s03900	*Pt-KAN.4*	3.27 ± 2.60	2.48 ± 0.85	0.5828
**POPTR_0003s09490**	***Pt-KAN2/3.1***	**8.34 ± 0.93**	**1.08 ± 0.29**	**2.281 × 10^−05^**
**POPTR_0001s02010**	***Pt-KAN2/3.2***	**5.48 ± 1.11**	**7.81 ± 2.46**	**0.1925**
**POPTR_0011s10070**	***Pt-PHB.1***	**27.16 ± 5.37**	**45.01 ± 8.31**	**0.0238**
**POPTR_0001s38120**	***Pt-PHB.2***	**25.69 ± 9.86**	**8.84 ± 1.02**	**0.0171**
**POPTR_0007s11880**	***Pt-PGY1.1***	**197.62 ± 39.30**	**99.77 ± 34.79**	**0.0174**
**POPTR_0001s45810**	***Pt-PGY2.1***	**61.44 ± 25.32**	**41.11 ± 19.57**	**0.2812**
**POPTR_0011s15170**	***Pt-PGY2.2***	**129.11 ± 57.05**	**63.09 ± 17.23**	**0.0746**
**POPTR_0013s13220**	***Pt-PGY3.1 ****	**406.38 ± 60.21**	**428.42 ± 107.56**	**0.7654**
**POPTR_0006s26980**	***Pt-RDR6.1 ****	**5.83 ± 2.12**	**15.81 ± 1.06**	**0.0004**
**POPTR_0018s01670**	***Pt-RDR6.2 ****	**1.13 ± 0.39**	**8.05 ± 2.57**	**0.0063**
**POPTR_0004s20730**	***Pt-SE.1***	**19.68 ± 2.93**	**24.42 ± 3.09**	**0.0956**
**POPTR_0009s16020**	***Pt-SE.2***	**25.06 ± 2.54**	**26.09 ± 5.93**	**0.7938**
**POPTR_0019s00300**	***Pt-SGS3.1***	**49.70 ± 3.60**	**44.12 ± 10.69**	**0.4333**
**POPTR_0001s07410**	***Pt-SGS3.2***	**20.06 ± 9.14**	**15.20 ± 15.20**	**0.3650**
POPTR_0001s07420	*Pt-SGS3.3*	0.27 ± 0.22	0.39 ± 0.07	0.3351
POPTR_0003s18660	*Pt-SGS3.4*	2.06 ± 0.65	1.84 ± 0.54	0.6411
POPTR_0003s18670	*Pt-SGS3.5*	0.30 ± 0.07	0.10 ± 0.04	0.0061
POPTR_0003s18680	*Pt-SGS3.6*	3.56 ± 0.07	2.99 ± 0.89	0.4061
POPTR_0003s18690	*Pt-SGS3.7*	0.41 ± 0.14	0.15 ± 0.06	0.0188
**POPTR_0003s01530**	***Pt-SGS3.8***	**2.22 ± 1.37**	**9.80 ± 6.01**	**0.0898**
**POPTR_0001s40870**	***Pt-SPL4.1 ****	**28.12 ± 3.72**	**60.65 ± 8.68**	**0.0019**
**POPTR_0011s11770**	***Pt-SPL4.2***	**9.61 ± 0.10**	**11.10 ± 7.10**	**0.7370**
**POPTR_0004s04630**	***Pt-SPL43.1***	**2.70 ± 0.96**	**9.59 ± 9.15**	**0.2605**
**POPTR_0011s05480**	***Pt-SPL43.2 ****	**51.31 ± 15.58**	**65.37 ± 24.85**	**0.4335**
**POPTR_0016s04890**	***Pt-SPL9.1***	**4.74 ± 1.95**	**9.24 ± 4.78**	**0.1914**
**POPTR_0001s22180**	***Pt-YAB2.1***	**102.90 ± 13.76**	**1.97 ± 1.53**	**2.352 × 10^−05^**
**POPTR_0127s00201**	***Pt-YAB2.2***	**16.82 ± 5.87**	**58.12 ± 30.25**	**0.0717**
**POPTR_0016s06760**	***Pt-YAB2.3***	**75.42 ± 16.33**	**64.62 ± 29.43**	**0.5962**
**POPTR_0003s11230**	***Pt-YAB3.1***	**61.42 ± 10.31**	**114.00 ± 51.14**	**0.1470**
**POPTR_0001s00240**	***Pt-YAB3.2 ****	**14.74 ± 2.50**	**66.94 ± 10.40**	**0.0004**
**POPTR_0006s06700**	***Pt-YAB5.1***	**47.58 ± 19.28**	**60.05 ± 2.61**	**0.2434**
**POPTR_0018s12990**	***Pt-YAB5.2***	**220.13 ± 18.32**	**0.01 ± 0.01**	**1.959 × 10^−06^**
POPTR_0006s26430	*Pt-YUC.2*	1.35 ± 0.63	1.08 ± 0.77	0.6348
POPTR_0018s01210	*Pt-YUC.1*	3.45 ± 1.59	1.27 ± 0.88	0.0652
POPTR_0006s26000	*Pt-YUC2.1*	2.91 ± 0.39	4.94 ± 1.81	0.1211
POPTR_0018s00840	*Pt-YUC2.2*	1.01 ± 0.67	0.76 ± 0.45	0.5844
POPTR_0003s11710	*Pt-ZPR1.1*	2.81 ± 1.35	2.76 ± 0.68	0.9491
POPTR_0001s08220	*Pt-ZPR1.2*	3.20 ± 1.47	1.47 ± 0.65	0.0845
POPTR_0002s15060	*Pt-ZPR2.1*	4.29 ± 3.00	3.35 ± 1.11	0.5780
**POPTR_0014s06690**	***Pt-ZPR2.2***	**17.75 ± 13.61**	**2.07 ± 1.45**	**0.0643**
**POPTR_0006s08320**	***Pt-ZPR3.1 ****	**18.88 ± 10.63**	**5.15 ± 1.65**	**0.0467**
POPTR_0010s24410	***Pt-ZPR3.2 ****	**0.47 ± 0.19**	2.62 ± 0.80	0.0066

**Table S3:** Mean Cq values (from qRT-PCR) used to calculate relative expression (aspen relative to black cottonwood) in leaf blades and petioles, graphed in Figure 3 and TIF5A control.

Gene name	Leaf blade			Leaf petiole		
	Expression difference	Mean Cq value in black cottonwood	Mean Cq value in aspen	Expression difference	Mean Cq value in black cottonwood	Mean Cq value in aspen
*Pt-AE7.2*	- *	32.28	34.69	- *	31.62	34.11
*Pt-AS1.1*	NS	35.01	34.54	- *	32.15	34.44
*Pt-AS1.2*	+ *	32.92	32.27	NS	34.04	33.81
*Pt-AS2.1*	- *	31.01	36.12	NS	36.30	35.90
*Pt-AS2.2*	NS	32.10	33.80	NS	34.10	36.37
*Pt-ATS.2*	- *	32.97	35.11	NS	36.71	36.53
*TIF5A*	NS	28.33	28.97	NS	27.07	28.39

- denotes lower transcript abundance or expression level in hybrid aspen or European aspen (for mRNA-seq data) in comparison to black cottonwood; + denotes higher transcript abundance or expression level in hybrid aspen or European aspen; asterisk (*) denotes significant difference in gene expression; NS – not significant.
